# Going beyond Learning and Listening to Meaningful Pursuit of Equity for Black Microbiologists

**DOI:** 10.1128/mBio.02821-20

**Published:** 2020-11-03

**Authors:** Emmanuel C. Adukwu, Musau WaKabongo, Okechukwu Ekenna, Bwalya Lungu, Amarachukwu Anyogu, Victor Ujor

**Affiliations:** a Department of Applied Sciences, University of the West of England, Frenchay Campus, Bristol, United Kingdom; b AIG, Co-Founder and Chief Financial Officer, President and Dr. Musau WaKabongo Science Education, Inc.; Retired County Public Health Laboratory/Director, California, USA; c Consultant in Infectious Diseases: Singing River Health System, Ocean Springs and Pascagoula, Mississippi, USA; d Department of Food Science and Technology, University of California, Davis, California, USA; e School of Life Sciences, University of Westminster, London, United Kingdom; f Bioenergy & Water Treatment Management Program, Centre for Applied Plant Sciences, The Ohio State University, Wooster, Ohio, USA; g African Initiative Group for Microbiology (AIG), affiliate of the American Society for Microbiology

## LETTER

We read the editorial on “The ASM Journals Committee values the contributions of Black microbiologists” published on 31 July 2020 (1) with interest and welcome both the acknowledgment of the barriers faced by underrepresented groups (URGs) in scientific publishing and actionable commitments to overcome these barriers. As highlighted in the editorial, “Black microbiologists should not have to shoulder the burden of dismantling systems of inequality on their own.” As microbiologists and regular contributors to American Society for Microbiology (ASM) conferences, ASM journals, and the global ASM community, we would like to offer additional comments and suggestions to the editorial. In response to the editorial from the Journals committee, we believe the following.

- Sustainable engagement that does not end at improving “recruitment” and “visibility” is imperative. As we have found out in higher education, recruitment without consideration for retention and success/progression is not a fail-safe strategy. Whittaker et al. (2) discuss the barriers that prevent increasing diversity in higher education institutions (HEIs) across the life cycle, many of which are applicable to scientific publishing such as “implicit bias” and “established environmental culture(s) and traditions.” Thus, a more sustainable strategy to achieve the goal of *recruiting*, *promoting*, and *ensuring retention* of Black microbiologists starts from improving access to contributing articles to the journals and participating in peer review activities which culminate in increased representation on journal committees and editorial boards is more sustainable.
- Diversity and inclusion without the necessary infrastructure will only paper over cracks. Many Black microbiologists work at institutions without the type of funding available at larger not-so-diverse institutions, which means these talented colleagues are unlikely to have the same levels of funding to compete at the level to produce the type of quality required in your journals. Thus, a financial model that does not continue to place a barrier where systemic barriers have existed for long periods is even more imperative. This could be in the form of article-processing charge (APC) waivers or exemptions, etc.
- Black microbiologists are not a homogenous community, and many existing members of ASM do not reside in the United States and come from different parts of the world, including lower- and middle-income countries (LMIC). These members, despite significant barriers of lack of representation and visibility, actively contribute to journals, attend the annual conferences at personal cost to themselves, and go out of their way to promote the Society. As an international society, the description of “Black microbiologists” should be extended to these communities also as they make significant contributions to the understanding and development of knowledge as well as global promotion of the society and the journals you lead. It is also important to recognize and acknowledge intersectionality and how race/ethnicity combine with other aspects of our individual identities in ways that hinder inclusivity (3). In this way, the goal to “leverage the experiences, perspectives, and expertise of everyone” would be achieved.
- We are particularly pleased to see mention of the Annual Biomedical Research Conference for Minority Students (ABRCMS) and the annual conference for the Society for Advancement of Chicanos/Hispanics & Native Americans in Science (SACNAS) in this editorial. We encourage more ASM members to engage with both meetings and encourage their students and colleagues to participate, supporting the development of young minority scientists and faculty who would benefit from broader engagement and recognition by others outside their own communities. In addition, we advocate for the journal editors to consider creating or repurposing a journal to publish some of the amazing work presented and shared at these two conferences. For many of the students and faculty who attend these meetings, this would be an important step in getting that first paper or manuscript.
- While we believe that the editorial is a step in the right direction, we also want to highlight that ASM has many microbiologists who are from Africa and the Diaspora. These microbiologists formed a group called the African Initiative Group (AIG) in 2003. Prior to the formation of this group, there was only one ambassador that covered all of Africa and the Middle East. For a continent this size, it was severely underrepresented. Through a few members raising their voice to the President of ASM, the AIG was formed. As a result of this, there are currently over two dozen Ambassadors. We applaud this although this is not far reaching. For the proposed suggestions to have any meaningful effect, it would require intentional approaches to recognize and amplify ASM members who are of/from Africa and the Diaspora; specialized funding to subgroups to enable progress in teaching, research, and developing research grant applications and publications in microbiology.
- Finally, we recommend consultations between the editors and the stakeholders from the Black microbiologists and discussions with members who roundtable with people who contribute manuscripts to the ASM journals to discuss their challenges as well sharing statistics of how many manuscripts are reviewed, returned, and published.

We commend the steps presented by the ASM Journals Committee to achieve equity within scientific publishing (1). Drawing from our lived experiences and the available research on initiatives to achieve equity in HEIs, consistent and transparent communication about progress achieving the cultural and structural change required is critical. As scientists, we follow the evidence. How will we evidence progress in achieving these commitments?

George Floyd, Breonna Taylor, and others we mourn do not represent only the Black American community, they represent a much wider community that are often voiceless, less represented, and unrecognized for the contributions made daily to advance our knowledge and understanding of microbiology.

“The journey of a thousand miles begins with one step.”—Lao Tzu. We welcome the steps mentioned in the editorial with the proposals made; hopefully, these steps will contribute to the much-needed change we want to see.
